# Urban and Suburban Differences in Hypertension Trends and Self-Care: Three Population-Based Cross-Sectional Studies from 2005-2011

**DOI:** 10.1371/journal.pone.0117999

**Published:** 2015-02-09

**Authors:** Gang Li, Huanhuan Hu, Zhong Dong, Jin Xie, Ying Zhou

**Affiliations:** 1 Institute of Chronic Diseases Control and Prevention, Beijing Center for Diseases Control and Prevention, Beijing, China; 2 Lab of Exercise Epidemiology, Graduate School of Sport Sciences, Waseda University, Saitama, Japan; University of Perugia, ITALY

## Abstract

**Objectives:**

This study aimed to compare hypertension trends in the urban and suburban population, and to examine the use of several self-care behaviors among patients who were aware of their hypertension.

**Methods:**

We examined the data from three cross-sectional adult populations obtained in 2005, 2008, and 2011, in Beijing.

**Results:**

Our analyses indicated that from 2005 to 2011 the standardized rate of hypertension increased from 31.9% to 36.0% (*P* <0.001) among urban adults, and was relatively stable (40.8% -40.2%) among suburban adults (*P* = 0.02). About 10% of the patients reported having taken measures to control their weight for hypertension management. As compared to the other patients, the female patients in the urban areas reported the highest rate of regular BP measurement (52.6%). In addition, the patients who reported taking medication regularly increased among the males and females. Most of the women reported nonsmoking (≥95%) and alcohol abstinence (≥90%). The trend of nonsmoking decreased among the urban males. In contrast, the prevalence of nonsmoking increased among the suburban males, though the trend was not statistically significant (*P* = 0.055). Further, the patient-reported alcohol abstinence was found to exhibit a decreasing trend among the males.

**Conclusions:**

We observed an increase in the hypertension prevalence from 2005 to 2011. The rates remained higher for suburban adults than for urban adults. Females generally had better self-care ability as compared to male patients. Further research is needed to promote self-care behaviors in hypertensive patients, especially for male patients.

## Introduction

Hypertension has been one of the most important public health challenges in the world. As of 2008, approximately 40% of adults aged ≥25 years have hypertension worldwide [1].In addition, the burden of hypertension is greater in low and middle income countries than in high income countries [1]. It is estimated that 230 million patients suffer from cardiovascular diseases, out of which 200 million have hypertension in China [2]. Evidence from recent systematic reviews and original reports indicated an increasing trend in the prevalence of hypertension in China [3,4,5,6]. Although there has been substantial research on the prevalence of hypertension, only a few large representative studies have compared the trends of hypertension between the urban and suburban, or rural population [4,5,6].

One approach that may improve blood pressure (BP) control and be feasible for the socioeconomically disadvantaged patients is patients’ involvement in their own care. Self-care behaviors have been documented as one of the main determinants of hypertension control [7]. Recently, more effort has been made to improve patients’ overall self-care [8,9]. Most of the research on self-care has been conducted in North America and Western Europe [10–14]. In China, studies assessing what activities individuals engage in to manage their BP, such as medication adherence, home BP monitoring, and exercise practices, are scarce [8,9]. It is vital to understand how Chinese patients manage hypertension through self-care behaviors because it can provide information for developing policies on support for self-care, suggest what practical actions can be taken, and provide ideas on how to support self-care.

Using data from the Beijing Chronic Disease Surveillance Project (BCDSP), we sought to provide updated findings on the trends of hypertension and self-care behaviors among hypertensive patients. Thus, in the present study, we analyzed data from three cross-sectional surveys of the BCDSP, which were conducted in 2005, 2008, and 2011. Our objectives were (1) to compare hypertension trends in the urban and suburban population, and (2) to examine the self-care behaviors of patients who were aware of their hypertension.

## Methods

The present study was approved by the Ethical Review Board of the Beijing Center for Disease Control and Prevention. Written informed consent was obtained from all participants prior to data collection. In addition, the participants were aware that they could stop the interview at any time and refuse to answer questions without having to provide a reason for the same.

### Study design

The BCDSP comprised a series of cross-sectional surveys that were designed to address the prevalence, distribution, trends, and risk factors of chronic diseases among adults aged 18–79 years in Beijing. It was initiated in 1999, by the Beijing Center for Disease Control and Prevention. Since then, this population-based surveillance has been carried out every three years. Beijing Municipality consists of six urban districts, eight suburban districts, and two rural counties, all of which were classified according to their location and economic development. The study sample was drawn from 14 urban and suburban districts, and two rural counties. About 1/850 of the adult population in Beijing were investigated in each survey. The number of participants selected from each district or county was proportional to its population.

A multistage, random cluster process was utilized to draw the survey sample. About 2/3^rd^ (before 2011)–3/4^th^ (in 2011) of the sample population was employed, and these participants were sourced from workplace. The unemployed participants were sampled from households in the residential committee/village. First, government offices provided a list of towns (names and population size) in each district and county. Then, the sample was drawn from the towns by utilizing the probability proportional to size (PPS) method. Thus, about 300 (before 2011)–400 (in 2011) people were selected from each sampled town. The number of surveyed towns equaled 1/850^th^ of the total adult population, divided by 300 or 400 people in each district and county. Second, 2–3 worksites and one residential committee/village were sampled from a list of worksites (name and population size) and communities (name and population size) in each of the selected towns utilizing the PPS sampling method. Third, about 100 people were sampled from each selected worksite using cluster random sampling, clustered by zone (floor or department). Fourth, one lane or one village was sampled from the selected community/village using PPS sampling method. Fifth, about 100 households were randomly selected from each sampled lane or village, and the Kish selection table was used for selecting an unemployed adult member within a selected household (including self-employed individuals and students aged 18 years or older).

Following this, the participants were asked to undergo a physical examination in local clinical centers and respond to a standardized questionnaire administrated by the investigators. The items in the questionnaire were divided into eight domains: socio-demographic characteristics, tobacco use, alcohol use, dietary habits, physical activity, weight control, health status, and family history. The staff involved in the study had received adequate training before the surveys, according to standard protocols.

### Participants

We analyzed data from three cross-sectional surveys conducted in 2005 (n = 16442), 2008 (n = 22301), and 2011 (n = 20242). All the three surveys used same protocols and similar questionnaires to collect data from the participants. The characteristics of the participants have been presented in Table 1.

**Table 1:** Characteristics of study participants in Beijing from 2005 to 2011.

	2005			2008			2011		
	Suburban	Urban	Total	Suburban	Urban	Total	Suburban	Urban	Total
Sample size, n (%)	6083 (37.0)	10359 (63.0)	16442 (100)	7700 (34.5)	14601 (65.5)	22301 (100)	7136 (35.4)	13106 (64.6)	20242 (100)
Female, n (%)	3466 (57.0)	6487 (62.6)	9953 (60.6)	4206 (54.7)	8259 (56.6)	12465 (56.0)	3926 (55.4)	7137 (54.9)	11063 (55.1)
Mean age, years (SE^d^)	43.7 (1.46)	46.1 (1.12)	45.3 (0.89)	41.7 (1.10)	45.3 (1.02)	44.0 (0.76)	43.7 (2.8)	45.5 (2.7)	44.8 (1.98)
Marital status, n (%)									
Married	-	-	-	6428 (83.5)	11806 (80.9)	18234 (81.8)	6149 (86.3)	10963 (83.6)	17112 (84.6)
Others	-	-	-	1272 (16.5)	2795 (19.1)	4067 (18.2)	987 (13.7)	2143 (16.4)	3130 (15.4)
Education level, n (%)									
≤6 years	965 (15.9)	1107 (10.7)	2072 (12.6)	896 (11.8)	1268 (8.8)	2164 (9.8)	702^**c**^ * (10.6)	872^**c**^ * (7.0)	1574^**b**^ * (8.2)
7–12 years	3006 (49.4)	5863 (56.6)	8869 (54.0)	4195 (54.6)	8176 (56.1)	12371 (55.6)	3513 (49.7)	8004 (61.2)	11517 (57.1)
>12 years	2112 (34.7)	3389 (32.7)	7151 (33.4)	2609 (33.6)	5157 (35.2)	7766 (34.6)	2921 (39.8)	4230 (31.9)	7151 (34.7)
Race, n (%)									
Han	5895 (96.9)	9859 (95.2)	15754 (95.8)	7385 (95.9)	13818 (94.6)	21203 (95.1)	6850 (96.1)	12369 (94.4)	19219 (95.0)
Others	188 (3.1)	500 (4.8)	688 (4.2)	315 (4.1)	783 (5.4)	1098 (4.9)	286 (3.9)	737 (5.6)	1023 (5.0)
BMI, n (%)									
BMI<18.5 kg/m2	158 (2.6)	279 (2.7）	437 (2.7)	141 (1.8)	325 (2.2）	466 (2.1)	145 (2.0)	301 (2.3）	446 (2.2)
18.5≤BMI<24.0 kg/m2	2315 (38.0)	4410 (42.5）	6725 (40.9)	2859 (37.0)	5868 (40.1）	8727 (39.0)	2454 (34.6)	4858 (37.4）	7312 (36.4)
24.0≤BMI<28.0 kg/m2	2303 (37.9)	3903 (37.7)	6206 (37.8)	2916 (37.9)	5637 (38.6)	8553 (38.4)	2733^**c**^ * (39.0)	5023^**c**^ * (39.0)	7756^**c**^ * (39.0)
BMI≥28.0 kg/m2	1305 (21.5)	1767 (17.1)	3072 (18.7)	1783 (23.2)	2771 (19.0)	4554 (20.5)	1687^**c**^ * (24.3)	2731^**c**^ * (21.2)	4418^**c**^ * (22.3)
Smoking, n (%)									
Female	169 (4.9)	333 (5.1)	502 (5.1)	121 (2.9)	278 (3.4)	399 (3.2)	93^**c**^ * (2.5)	254^**a**^ ^**c**^ * (3.6)	347^**b**^ * (3.2)
Male	1609 (61.5)	2161^**a**^ * (55.8)	3770 (58.1)	2120 (60.7)	3460^**a**^ * (54.5)	5580 (56.7)	1861^**c**^ * (58.0)	3445^**c**^ * (57.4)	5306 (57.6)
Drinking, n (%)									
Female	338 (9.7)	698 (10.8)	1036 (10.4)	334 (7.9)	730 (8.8)	1064 (8.5)	406 (10.3)	714 (9.8)	1120 (10.0)
Male	1783 (68.1)	2348^**a**^ * (60.6)	4131 (63.6)	2165 (61.9)	3550^**a**^ * (56.0)	5715 (58.1)	2149 (66.8)	3487^**a**^ * (58.1)	5636^**b**^ * (61.2)

* Significant at *P* < 0.05.

^a^ Significant difference between suburban population and urban population within each survey.

^b^ Cochran-Armitage trend test for overall prevalence

^c^ Cochran-Armitage trend test for prevalence within suburban participants or urban participants

- Data were not available.

^d^ SE Standard error

### Measures

The following variables were selected from the BCDSP dataset.

### Socio-demographic characteristics

The socio-demographic variables included in the present study were gender, age, educational level (in terms of years of education, specifically, ≤ 6 years, 7–12 years, and >12 years), race (Han and others), and marital status. The area of residence was classified as urban (eight districts in the downtown), and suburban (this included the rural areas because from the BCDSP dataset we could not correctly identify and exclude the participants who lived in the rural villages).

### Anthropometric measurements

Height and weight were measured by trained health workers who followed standard protocols. Weight was measured in lightweight clothing, to the nearest 0.1 kg, and height was measured to the nearest 0.1 cm. The body mass index (BMI) was calculated from the weight and height. Further, the BMI (kg/m2) was categorized according to the Chinese criteria, as underweight (BMI < 18.5), normal weight (18.5 ≤ BMI < 24), overweight (24≤BMI<28), and obese (BMI ≥ 28) [15].

### BP

The BP was measured thrice in a sitting position after at least 5 minutes of rest, by using a standardized digital BP measuring machine (Omron Digital HEM-770A). The second and third BP readings were averaged. In the present study, hypertension was defined as an average systolic BP (SBP) ≥140 mm Hg, an average diastolic BP (DBP) ≥ 90 mm Hg, and/or self-reported current treatment for hypertension with anti-hypertensive medication. Similarly, awareness of hypertension was defined as a self-report of any prior medical diagnosis of hypertension. In addition, BP control among persons with hypertension was defined as an average SBP < 140 mm Hg and an average DBP < 90 mm Hg.

### Self-care behaviors

Participants who did not smoke were considered non-smokers. With reference to alcohol intake, participants who reported no alcohol consumption were considered abstainers. Participants who reported taking their antihypertensive medications as prescribed by the physician were considered adherent, while all others were considered non-adherent. With reference to regular BP measurement, patients who reported measuring BP regularly were considered to be adherent. Participants were considered to exhibit weight management behavior if they reported to have taken measures (e.g., physical exercise, healthy diet) to control weight in the past 12 months. Items about regular BP measurement and weight management were added in the 2011 survey.

### Statistical analysis

All statistical analyses were performed using SAS software, version 9.2 (SAS Institute, Cary, NC). Descriptive statistics were generated with sample size, percentage, and mean. The Chi-square and Cochran-Armitage trend tests were used where appropriate. Similar to previous reports, the survey design parameters including weight, stratum, and cluster were incorporated into all analysis [16,17]. All estimates were weighted; the weights were derived based on the population composition in each survey year. PROC SURVEYFREQ and PROC SURVEYMEANS were used for the calculation of prevalence and mean. The overall prevalence, awareness, and control rates of hypertension were adjusted for age and gender by direct standardization to the total population of the three surveys (n = 58985). The values were considered to be statistically significant at *P* = 0.05.

## Results

### Characteristics of the study participants

The characteristics of the study participants from 2005 to 2011 have been presented in Table 1. The average age of the sample was about 44 years. The proportion of participants who had education ≤ 6 years decreased from 12.6% in 2005 to 8.2% in 2011 (*P* < 0.001). More than 95% of the participants were Han Chinese. The proportion of overweight and obese participants increased from 56.5% in 2005 to 61.3% in 2011 (*P* < 0.001). The participants who reported to indulge in smoking ranged between 58.1% in 2005 and 57.6% in 2011 among males (*P* = 0.85), and was found to have decreased among females, from 5.1% in 2005 to 3.2% in 2011 (*P* < 0.001). In contrast, male participants exhibited a reduction in the tendency to consume alcohol, evident from a decrease from 63.6% in 2005 to 61.2% in 2011 (*P* < 0.03). As for female participants, it ranged between 10.4% in 2005 and 10.0% in 2011 (*P* = 0.57). The prevalence of alcohol and tobacco use among suburban males was higher than that among urban males. However, there was no significant difference in the prevalence of alcohol and tobacco use between urban and suburban females (Table 1).

Trends in prevalence, awareness, and control of hypertension from 2005 to 2011

Table 2 presents the trends related to prevalence, awareness, and control of hypertension among participants, from 2005 to 2011. Overall, the participants with hypertension increased from 35.2% in 2005 to 37.4% in 2011 (*P* < 0.001). Further, the age and gender adjusted prevalence of hypertension increased from 31.9% in 2005 to 36.0% in 2011 (*P* < 0.001) among the urban adults, and it ranged between 40.8% in 2005 and 40.2% in 2011 among the suburban adults (*P* = 0.02). Moreover, in contrast to the urban adults, the suburban participants had a higher prevalence of hypertension. The standardized prevalence of awareness among the hypertensive patients increased moderately from 40.9% in 2005 to 42.7% in 2011 (*P* < 0.001). In addition, the proportion of the suburban patients reporting awareness of hypertension appeared comparable to that in the urban patients. The standardized control rate of hypertension increased from 9.4% to 14.0% (*P* < 0.001) over the 6-year period. It was observed that the patients in the urban areas had better hypertension control than those in the suburban areas.

**Table 2:** Prevalence, awareness, and control of hypertension among adults in Beijing from 2005 to 2011.

	2005			2008			2011^e^		
	Suburban	Urban	Total	Suburban	Urban	Total	Suburban	Urban	Total
Prevalence n (%, SE^d^)	2406/6083 (39.6, 2.84)	3499/1035 (33.8, 2.15)	5905/1644 (36.0, 1.72)	2914/7700 (37.8, 2.55)	5350/1460 (36.8, 1.89)	8264/2230 (37.2, 1.51)	2652/7016 (38.6, 5.21)	4760/1292 (37.5, 4.11)	7412/1994 (37.9, 3.26)
18–34	206/1684 (12.2, 1.17)	188/2295 (8.2, 0.87)	394/3979 (9.9, 0.72)	356/2412 (14.8, 1.70)	435/3549 (12.3, 1.13)	791/5961 (13.3, 0.95)	271/2065 (13.1, 1.14)	410/3311 (12.4, 1.40)	681/5376 (12.7, 0.99)
35–44	531/1634 (32.5, 1.82)	519/2496 (20.8, 1.30)	1050/4130 (25.4, 1.11)	787/2394 (32.9, 1.85)	859/3338 (25.8, 1.41)	1646/5732 (28.8, 1.13)	566/1969 (28.8, 1.57)	788/2988 (26.4, 1.12)	1354/4957 (27.3, 0.89)
45–54	793/1547 (51.3, 1.95)	1205/3194 (37.7, 1.31)	1998/4741 (42.2, 1.14)	919/1727 (53.2, 1.50)	1857/4330 (42.9, 1.06)	2776/6057 (45.9, 0.94)	844/1671 (50.6, 1.67)	1821/3941 (46.2, 1.08)	2665/5612 (47.6, 0.89)
55–64	516/750 (68.8, 1.95)	666/1158 (57.5, 1.60)	1182/1908 (62.0, 1.30)	477/686 (69.6, 2.01)	1207/2050 (58.9, 1.70)	1684/2736 (61.6, 1.44)	570/822 (69.5, 2.10)	1008/1686 (60.0, 2.62)	1578/2508 (63.2, 1.94)
65–79	360/468 (76.9, 2.43)	921/1216 (75.7, 1.50)	1281/1684 (76.1, 1.28)	375/481 (78.0, 2.65)	992/1334 (74.4, 1.36)	1367/1815 (75.3, 1.2)	401/489 (82.0, 1.65)	733/1002 (73.2, 1.19)	1134/1491 (76.1, 1.41)
Total^s^, %	40.8	31.9^a^ *	35.2	42.0	35.2^a^ *	37.4	40.2^c^ *	36.0^a^ ^c^ *	37.4^b^ *
Awareness n (%, SE)	1154/2406 (48.0, 1.95)	1770/3499 (50.6, 1.60)	2924/5905 (49.5, 1.23)	1251/2914 (42.9, 1.74)	2557/5350 (47.9, 1.36)	3808/8264 (46.1, 1.09)	1336/2652 (50.7, 2.81)	2459/4760 (52.2, 3.71)	3795/7412 (51.7, 2.54)
18–34	46/206 (22.3, 3.21)	26/188 (13.8, 3.12)	72/394 (18.3, 2.25)	67/289 (18.8, 2.62)	67/435 (15.4, 2.23)	134/791 (17.0, 1.69)	43/271 (15.9, 2.61)	81/410 (19.7, 1.39)	124/681 (18.2, 1.61)
35–44	198/531 (37.3, 2.51)	200/519 (38.5, 2.35)	398/1050 (37.9, 1.72)	291/787 (37.0, 2.40)	293/859 (34.1, 1.81)	584/1646 (35.5, 1.49)	255/566 (44.9, 2.42)	305/788 (38.8, 1.70)	560/1354 (41.4, 1.55)
45–54	404/793 (51.0, 2.56)	649/1205 (53.9, 2.16)	1053/1998 (52.7, 1.63)	447/919 (48.6, 2.18)	897/1857 (48.3, 1.46)	1344/2776 (48.4, 1.21)	455/844 (53.8, 1.87)	937/1821 (51.5, 2.23)	1392/2665 (52.2, 1.71)
55–64	307/516 (59.5, 2.36)	384/666 (57.7, 2.18)	691/1182 (58.5, 1.61)	244/477 (51.1, 2.09)	675/1207 (55.9, 1.80)	919/1684 (54.6, 1.44)	342/570 (59.9, 2.12)	619/1008 (61.5, 1.67)	961/1578 (60.9, 1.33)
65–79	199/360 (55.3, 3.04)	511/926 (55.5, 2.03)	710/1281 (55.4, 1.69)	202/375 (53.9, 3.10)	625/992 (63.0, 1.93)	827/1367 (60.5, 1.71)	241/401 (60.1, 2.60)	517/733 (70.5, 2.62)	758/1134 (66.8, 2.58)
Total^s^, %	41.4	40.2^a^ *	40.9	38.6	38.6	38.8	42.5^c^ *	42.9^c^ *	42.7^b^ *
Control n(%, SE)	193/2406 (8.0, 0.90)	446/3499 (12.7, 1.01)	639/5905 (10.8, 0.69)	226/2914 (7.7, 0.69)	735/5350 (13.7, 0.76)	961/8264 (11.6, 0.59)	330/2652 (12.4, 0.81)	836/4760 (17.8, 1.66)	1166/7412 (15.8, 0.95)
18–34	5/206 (2.4, 0.99)	7/188 (3.7, 1.73)	12/394 (3.0, 0.98)	13/356 (3.6, 1.13)	19/435 (4.4, 1.24)	32/791 (4.0, 0.86)	8/271 (2.9, 0.92)	29/410 (7.1, 0.88)	37/681 (5.4, 0.75)
35–44	28/531 (5.3, 0.97)	61/519 (11.8, 1.70)	89/1050 (8.5, 0.99)	39/787 (4.9, 1.05)	83/859 (9.7, 1.16)	122/1646 (7.4, 0.80)	74/566 (12.9, 1.31)	105/788 (13.4, 1.88)	179/1354 (13.2, 1.22)
45–54	79/793 (10.0, 1.37)	219/1205 (18.2, 1,82)	298/1998 (14.9, 1.21)	97/919 (10.5, 1.03)	280/1857 (15.0, 1.22)	377/2776 (13.5, 0.90)	123/844 (14.4, 1.27)	303/1821 (16.7, 1.37)	425/2665 (16.0, 0.93)
55–64	61/516 (11.8, 2.16)	86/666 (12.9, 1.60)	147/1182 (12.4, 1.30)	47/477 (9.8, 1.28)	233/1207 (19.3, 1.22)	280/1684 (16.6, 1.03)	78/570 (13.5, 1.64)	240/1008 (23.8, 1,11)	318/1578 (20.1, 1.15)
65–79	20/360 (5.6, 0.74)	73/921 (7.9, 1.01)	93/1281 (7.3, 0.76)	30/375 (8.0, 1.47)	120/992 (12.1, 1.14)	150/1367 (11.0, 0.96)	47/401 (11.7, 2.13)	159/733 (21.6, 1.95)	206/1134 (18.1, 1.96)
Total^s^, %	6.8	11.6^a^ *	9.4	7.3	11.7^a^ *	9.4	11.1^c^ *	16.0^a^ ^c^ *	14.0^b^ *

* Significant at *P* < 0.05.

^a^ Significant difference between suburban population and urban population within each survey.

^b^ Cochran-Armitage trend test for overall prevalence.

^c^ Cochran-Armitage trend test for prevalence within suburban participants or urban participants.

^d^ SE Standard error.

^e^ 298 participants with missing BP values were excluded.

^s^ Age and gender adjusted using the total population (n = 58985).

Self-care behaviors among patients who were aware of their hypertension

Table 3 shows the self-care behaviors among the patients who were aware of their hypertension. Significant differences in the proportion of participants engaging self-care behaviors existed between genders within the urban areas or suburban areas. About 10% of the patients reported having taken measures to control their weight for hypertension management. As compared to the other patients, the female patients in the urban areas reported the highest rate of regular BP measurement (52.6%). In addition, the patients who reported taking medication regularly increased from 26.9% in 2005 to 62.8% in 2011 among the suburban males (*P* < 0.001), and from 30.5% in 2005 to 73.9% in 2011 among the urban females (*P* < 0.001). Similar trends were also found among the urban females and suburban females. Most of the women reported nonsmoking (≥ 95%) and alcohol abstinence (≥ 90%). However, the trend of nonsmoking decreased from 50.5% in 2005 to 43.6% in 2011 among the urban males (*P* < 0.001). In contrast, the prevalence of nonsmoking increased from 39.5% in 2005 to 45.1% in 2011 among the suburban males, though the trend was not statistically significant (*P* = 0.055). Further, the patient-reported alcohol abstinence was found to exhibit a decreasing trend, from 34.2% in 2005 to 29.7% in 2011 among the suburban males *(P* = 0.040), and from 45.2% in 2005 to 39.2% in 2011 among the urban males (*P* < 0.001). Overall, the prevalence of alcohol abstinence was higher in the urban males than that among the suburban males.

**Table 3:** Self-care behaviors among patients who were aware of their hypertension in Beijing from 2005 to 2011.

			Suburban		Urban	
			Male	Female	Male	Female
2011			N = 658	N = 678	N = 1280	N = 1179
	Self-care behaviors, n(%, SE^d^)					
		Regular BP measurement	230 (35.0, 1.62)	271 (40.0, 1.73)	481 (38.1, 2.90)	615 (52.6, 3.17)^a^ ^b^ *
		Weight control	77 (11.4, 2.11)	51 (7.3, 1.56)^a^ *	117 (9.1, 1.02)	116 (9.8, 0.83)
		Medication adherence	410 (62.8, 3.06)^c^ *	498 (73.9, 3.20)^a^ ^c^ *	824 (65.0, 3.68)^c^ *	910 (77.8, 3.79)^a^ ^c^ *
		Non-smoking	296 (45.1, 2.77)	644 (95.0, 1.64)^a^ ^c^ *	549(43.6, 4.35)^c^ *	1121 (95.1, 0.44)^a^ *
		Alcohol abstinence	191 (29.7, 3.73)^c^ *	623 (91.9, 1.07)^a^ *	493 (39.2, 4.09)^b^ ^c^ *	1086 (92.4, 2.19)^a^ *
2008			N = 638	N = 613	N = 1139	N = 1418
	Self-care behaviors, n(%, SE)					
		Medication adherence	315 (49.4, 2.80)	378 (61.8, 3.47)^a^ *	597 (52.5, 2.42)	923 (65.2, 2.27)^a^ *
		Non-smoking	271 (42.5, 2.01)	591 (96.4, 0.86)^a^ *	530 (46.7, 2.60)	1360 (95.9, 0.62)^a^ *
		Alcohol abstinence	235 (36.9, 2.72)	583 (95.1, 0.86)^a^ *	482 (42.5, 2.49)^b^ *	1319 (93.0, 0.81)^a^ *
2005			N = 547	N = 607	N = 713	N = 1057
	Self-care behaviors, n(%, SE)					
		Medication adherence	147 (26.9, 2.07)	185 (30.5, 3.13)	196 (27.5, 1.89)	280 (26.5, 2.46)
		Non-smoking	216 (39.5, 2.56)	559 (92.1, 1.06)^a^ *	360 (50.5, 3.28)^b^ *	989 (93.6, 0.89)^a^ *
		Alcohol abstinence	187 (34.2, 3.52)	563 (92.8, 1.17)^a^ *	322 (45.2, 3.19)^b^ *	972 (92.0, 0.91)^a^ *

* Significant at *P* < 0.05.

^a^ Significant difference between genders within urban patients or suburban patients.

^b^ Significant difference between urban patients and suburban patients for each gender.

^c^ Cochran-Armitage trend test for each gender by urban or suburban group.

^d^ SE Standard error.

## Discussion

In this study, we examined the trends related to the prevalence, awareness, and control of hypertension among urban and suburban hypertensive patients. In addition, we compared the self-care behaviors of the patients from these two areas.

Overall, the study showed a significant increase in hypertension prevalence among adults in Beijing. This has also been reported by other recent studies on Chinese adults [3,6]. The increasing prevalence of hypertension has been attributed to population growth, aging and unhealthy behaviors, such as smoking, harmful use of alcohol, physical inactivity, and excess weight [1]. In the present study, the proportion of overweight and obese persons increased from 56.5% in 2005 to 61.3% in 2011 (*P* < 0.001). This may partly explain the increase in the prevalence of hypertension.

The rate of hypertension in the urban adults increased moderately from 31.9% to 36.0% (*P* < 0.001), whereas that for the suburban adults seemed relatively stable, with a slight decrease (from 40.8% to 40.2%, *P* = 0.02). Further, despite the differences in the trends of hypertension prevalence, the rates remained higher for the suburban adults than for the urban adults. So far, there is conflicting evidence about the disparity of hypertension prevalence among urban and suburban/rural areas [4,5]. A recent study suggested that population in the urban areas had higher prevalence of hypertension than in the rural areas [6]. However, as these studies differed in terms of the sample characteristics, survey periods, and other potential determinants, comparisons were impossible.

Lack of hypertension awareness and control is a major problem in developing countries [18,19]. The rates of hypertension awareness and control in the United States and Canada have improved to > 80% and > 50%, respectively [20,21]. In contrast, more than half of the hypertensive patients in the present study were still found to be unaware of their condition, and about 10% of the patients were able to control their BP. Therefore, the hypertension awareness and control rates observed in the present study continue to cause concern.

In the current study, we found significant gender differences in the proportion of participants engaging self-care behaviors within the urban and suburban areas. This finding is consistent with that of previous studies [8,10, 22]. Further, the rates were much lower for self-care behaviors related to measuring one’s BP regularly and managing or losing weight. This may be due to the low knowledge of BP monitoring and weight control among the patients. On the other hand, we found a rapid increase in medication adherence among the patients who were aware of their condition from 2005 to 2011. This may be due to the aggressive marketing of antihypertensive drugs in China [23]. Noticeably, the control rate of hypertension did not increase as fast as medication adherence did. Similar results were reported by another study [17]. However, it is important to acknowledge that the control of hypertension is not only dependent on medication, but it also involves other factors (e.g., lifestyles, mental health) [24,25]. In addition, we also should caution that the medication adherence maybe over reported by the patients. For example, response bias can occur due to the respondent’s tendency to over-report good behavior.

Table 3 showed that less than half of the males with hypertension reported abstinence from alcohol and tobacco use across the three surveys. The difference between the males in the suburban and urban areas in terms of their nonsmoking rate was significant in 2005 and 2008; however, it was found to have disappeared in 2011. This may be caused by the increase in the urban males’ tendency for smoking from 2005 to 2011. In contrast, the rates of alcohol abstinence decreased among both suburban and urban males during this period. However, the reasons for these changes are not clear. Further surveillance data may be needed to clarify these changes. The findings of this study indicated that it is still a big public health challenge to promote smoking cessation and alcohol abstinence among male hypertensive patients. Therefore, more efforts may be needed for male patients.

### Limitations

This study, however, has several limitations. First, the period for examining the trends in hypertension is relatively short. Second, the data on self-care in this study were obtained through a self-report questionnaire, and therefore, recall bias was inevitable. Third, The BCDSP has been conducted since 1995, however, the survey questions changed with the accumulation of scientific evidence on chronic diseases. Finally, we used our own criteria to assess self-care behaviors. The results may be affected by the lack of established adherence to the criteria.

## Conclusions

The current study found that about one in third adults in Beijing had hypertension. We observed an increase in the hypertension prevalence from 2005 to 2011. The rates remained higher for suburban adults than for urban adults. Although the awareness and control rates of hypertension improved slightly in both urban and suburban patients with hypertension, the levels of hypertension awareness and control were still very low. More efforts are required to promote the control of hypertension. Females generally had better self-care ability as compared to male patients. Further research is needed to promote self-care behaviors in hypertensive patients and to ensure the quality of self-care, especially for male patients.

## Supporting Information

S1 Dataset Detailed data of this paper. (CSV)
